# “Convenience, Quickness, and Compassion”: Experiences of People Involved in the Criminal‐Legal System Accessing Medications for Opioid Use Disorder Services From a Mobile Unit in Chicago

**DOI:** 10.1111/hex.70552

**Published:** 2026-01-06

**Authors:** Toni Martinford, Dennis P. Watson, Sarah Messmer, Leyla Rashid, Monte Staton, Michael L. Dennis, Christine E. Grella, Abigail Elmes‐Patel

**Affiliations:** ^1^ Department of Pharmacy Practice University of Illinois Chicago Retzky College of Pharmacy Chicago Illinois USA; ^2^ Lighthouse Institute Chestnut Health Systems Chicago Illinois USA; ^3^ Department of Medicine University of Illinois Chicago College of Medicine Chicago Illinois USA; ^4^ University of Illinois Chicago Institute for Research on Addictions Chicago Illinois USA

## Abstract

**Background:**

This study aimed to explore the experiences of people diagnosed with opioid use disorder (OUD) and recently involved with the criminal‐legal system (CLS) as they received addiction treatment services from a Mobile Medications for Opioid Use Disorder (MOUD) programme in Chicago. By assessing perceptions and satisfaction with receiving addiction treatment and additional wraparound services, including factors influencing care‐seeking decisions, this research provides insight to tailor care and services to this population of people who have involvement with the CLS. Insights from this population are critical to increasing their access to MOUD and other services, given that people with CLS involvement are at disproportionately higher risk of overdose than people without such involvement.

**Methods:**

Semi‐structured interviews were conducted with people with OUD engaged in mobile MOUD services who had CLS involvement in the past 90 days (arrested, booked, or charged; released from prison, jail, or electronic monitoring; or currently on probation, parole, or supervised release). Eligibility for participation was determined by an outreach specialist with knowledge of past service utilisation and access to the medical record. Questions were based on Consolidated Framework for Implementation Research (CFIR) domains. Interviews were recorded, transcribed, deidentified, and inductively analyzed using Dedoose qualitative analysis software. The primary phenomenon of interest was a description of perceived benefits, barriers, and facilitators to accessing mobile MOUD services by CLS‐involved individuals.

**Results:**

A total of 13 interviews were conducted. The mean age of the participants was 48.5 years (SD 11.4). Over half of participants identified as Black or African American (61.5%) and male (61.5%). In the 30 days prior to interview, seven (53.8%) participants reported stable housing, five (38.5%) were living outdoors, and one (7.7%) had been incarcerated. Five themes and eight sub‐themes were identified. Participants viewed the mobile unit services as essential to their communities, citing proximity to an open‐air drug market and limited services in the area. Participants overwhelmingly praised the non‐judgmental, approachable, and trustworthy environment compared to other healthcare settings. Law enforcement interactions ranged from positive to neutral, with some noting CLS referrals to mobile medical unit care. Key facilitators included onsite MOUD dispensing, walk‐in appointments, and comprehensive medical services. Word‐of‐mouth, co‐utilisation of mobile services with partners, friends, and/or family, and intrinsic self‐motivation were identified as facilitators to mobile unit access. Barriers to accessing care included incarceration, transportation, and schedule/service awareness.

**Conclusions:**

By exploring the perspectives of people with opioid use disorder and involvement with the CLS, the study contributes to the design and tailoring of an established mobile MOUD programme to meet their collective needs, including broader implementation of services that support re‐entry to the community, like peer recovery and recovery‐oriented support groups.

**Patient Contribution:**

This study directly involved people with opioid use disorder who were recently involved in the criminal‐legal system, a population historically marginalised in healthcare settings, enabling a client‐informed approach to service improvement. By centreing their voices through qualitative interviews, the research provides insight into how structural barriers such as incarceration, housing instability, stigma, and limited service availability affect their ability to access treatment. Results from this qualitative study will be discussed with members of the Community Outreach Intervention Projects (COIP) organisation who have lived experience with substance use disorders and living experience with the neighbourhoods these services are offered in. The findings inform actionable strategies to adapt mobile healthcare services to better meet the unique needs of this population, including facilitating access immediately post‐release.

## 1 Introduction

Synthetic opioids accounted for 48,422 US deaths in 2024 [1]. Fatal drug overdose remains the leading cause of death for those ages 18–44 [2], despite a sharp decrease in overdose deaths nationwide [1]. Black and Hispanic communities have been disproportionately impacted by opioid overdose deaths (OOD) and experience decreased access to medications for opioid use disorder (MOUD)—e.g., methadone, buprenorphine, and long‐acting, injectable naltrexone [3]. In addition to an increased rate of opioid use disorder (OUD), Black and Hispanic people are up to six times more likely to experience incarceration than White people [4]. This injustice, fuelled by the decades‐old War on Drugs, over‐policing of communities of colour, and discriminatory practices of the criminal‐legal system (CLS), has resulted in far more drug‐related incarcerations for Black and Hispanic people than White people, despite similar rates of drug use amongst those groups [5]. Given these data, it is no surprise that people involved with the CLS are more likely to have an OUD than the general public [6, 7].

Medications for OUD in particular buprenorphine and methadone, reduce opioid overdose risk and acute treatment for serious events related to opioids [8]. Despite the known efficacy of MOUD, it is estimated that only 1 in 5 people with OUD receive it [7]. Barriers to MOUD access and treatment retention are extensive and include legal and regulatory challenges, housing instability, public insurance pitfalls, inadequate professional training in both the medical and judicial systems, stigma, and concerns about medication diversion [9, 10]. Those with CLS involvement face additional barriers to accessing MOUD. They experience gaps in healthcare insurance during reentry [11], endure stigma toward MOUD from personnel working in the CLS [12], are more likely to be unhoused prior to entering jail or prison, and face additional challenges obtaining housing upon reentry [13]. Only 5% of people in prison or jail receive these life‐saving medications while incarcerated, and only 33.6% of those referred to OUD treatment by the CLS receive MOUD as part of their care plan [14, 15]. In part due to decreased access to MOUD, people with a history of incarceration have higher rates of overdose and OOD, especially immediately following release [16, 17, 18]. Given their disproportionate risk of overdose upon reentry, it is imperative to quickly connect people who have recently been involved in the CLS and have OUD with these lifesaving medications.

Mobile medical units offering low‐threshold buprenorphine have been implemented in multiple cities in the United States to address some of the multifaceted barriers to accessing MOUD [19, 20]. These units facilitate interdisciplinary healthcare teams to integrate addiction treatment, medical, and a multitude of other social and health‐related services directly into high need areas, such as outside of an urban county jail or near known opioid overdose hot spots. Offering walk‐in appointments and same‐day buprenorphine prescribing has been shown to increase retention in treatment and improve access to care, especially for marginalised groups such as Black people and those with unstable housing [20, 21, 22].

### 1.1 Current Study

In 2021, the Community Outreach Intervention Projects (COIP) began a Mobile MOUD programme in collaboration between the University of Illinois Chicago (UIC) College of Medicine, College of Pharmacy, and School of Public Health. The unit provides low‐threshold buprenorphine and referrals to methadone programmes, along with comprehensive healthcare and additional wraparound services, to people who use drugs in the West Side of Chicago, a region considered an open drug market with consistently and disproportionately high opioid overdose rates [23, 24]. Wraparound services include wound care, harm reduction supply distribution, provision of food and clothing donations as available, and linkage to addiction treatment services such as methadone, in‐patient substance use programmes, and brick‐and‐mortar clinics if preferred. The MOUD team comprises at least one outreach worker, a physician, and a clinical pharmacist, and is augmented by various health professional trainees. The Mobile MOUD programme operates with a harm reduction approach, emphasising non‐judgmental, non‐coercive services that minimise drug‐related harms [25].

In alignment with the Indigenous Leader Outreach Model [26], COIP employs team members with lived experience (of substance use) and living experience (with the neighbourhoods) in a multitude of roles including recovery support specialists and outreach specialists. Lived experience refers to people who have experienced something (such as substance use) in the past, whereas living experience denotes a current experience [27]. From July 2021 to December 2024, the Mobile MOUD programme saw 1581 unique patients over 4378 encounters. Of those, 998 patients were seen for buprenorphine initiation and management across 3316 visits and received 1559 pre‐made buprenorphine packs [19]. The mobile unit welcomes new and returning patients for buprenorphine initiation, follow‐up, and if needed, reinitiation.

Our goal was to qualitatively explore the experiences of people diagnosed with OUD and recently involved with the CLS as they received addiction treatment services and/or additional wraparound services from the COIP Mobile MOUD programme. We specifically aimed to assess their perceptions and satisfaction with receiving services from the unit, including internal and external factors influencing their decision to seek care, to ultimately tailor services to this population. To achieve our target, we consulted various determinant frameworks, which are theoretical approaches used in implementation science, in the creation of our interview guide. Implementation science emphasises the importance of understanding multi‐level influences to appropriately assess the mechanisms by which evidence‐based practice implementation is most likely to succeed [28]. This approach allowed for us to identify facilitators and barriers to effective process implementation by gathering information from people who have influence over outcomes, such as clients directly receiving healthcare services.

## 2 Materials and Methods

People with CLS involvement in the past 90 days (arrested, booked, or charged; released from prison, jail, or electronic monitoring; or currently on probation, parole, or supervised release) and OUD engaged in COIP mobile MOUD and wraparound services were eligible for a 30‐min semi‐structured interview. The interview guide (Table 1) was developed with questions focused on domains commonly recognised as affecting implementation processes and outcomes, predominantly the Consolidated Framework for Implementation Research (CFIR) [27]. Subjects were recruited from people accessing COIP mobile units and clinics via flyers and verbal recruitment by an outreach specialist. Eligibility was determined by the outreach specialist with access to the medical chart, who would ask mobile unit clients known to have OUD and multiple encounters with the unit if they had recent experience with the CLS. A $50 Master Card gift card was offered for participation.

**Table 1:** Interview question guide.

Intervention characteristics
1. Can you describe your first impression of the buprenorphine mobile unit? What were your initial thoughts or concerns? (Intervention Source)
2. How does the mobile unit differ from other health services you have used in the past? (Relative Advantage)
3. What do you think about the convenience of accessing the mobile unit? (Adaptability)
4. What features of the mobile unit do you find most helpful, and why? (Complexity)
5. Were there any aspects of the mobile unit that were confusing or difficult to use? How did you handle that? (Complexity)
Outer setting
6. What external factors (e.g., weather, location, distance) influence your decision to use the mobile unit? (Patient Needs & Resources)
7. How does your relationship with law enforcement or other legal authorities affect your ability to use the mobile unit? (External Policy & Incentives)
8. Are there any community resources or organisations that have supported your use of the mobile unit? (Peer Pressure)
Inner setting
9. How welcoming and supportive do you find the environment of the mobile unit? (Culture)
10. Can you describe any support you receive from family, friends, or peers in accessing the mobile unit? (Network & Communications)
11. Have you faced any logistical challenges, like timing or transportation, when trying to access the mobile unit? (Implementation Climate)
Characteristics of individuals
12. What personal factors or experiences motivated you to use the mobile unit for buprenorphine treatment? (Knowledge & Beliefs about the Intervention)
13. How confident do you feel about using the mobile unit to manage your treatment? (Self‐efficacy)
14. Have you encountered any personal barriers, such as stigma or privacy concerns, that affected your use of the mobile unit? (Individual Stage of Change)
15. Can you share any personal successes or challenges you've experienced while using the mobile unit? (Individual Identification with Organisation)
Process
16. What was your experience like the first time you used the mobile unit? (Engaging)
17. How do you think the mobile unit staff could improve the services they offer? (Executing)
18. Are there any changes or improvements you would like to see in how the mobile unit operates? (Reflecting & Evaluating)
19. How do you stay informed about the services and schedules of the mobile unit? (Planning)
20. Can you describe any feedback you have provided to the mobile unit staff and how they responded? (Engaging)
Additional questions
21. How do you perceive the role of the mobile unit in your overall health and recovery journey? (Overall Experience)
22. What factors would make you more likely to continue using the mobile unit for treatment? (Sustainability)
23. Can you share any specific stories or experiences that highlight the impact of the mobile unit on your life? (Personal Experience)
Conclusion
24. Is there anything else you would like to add about your experiences with the buprenorphine mobile unit? (General Feedback)

Interviews were conducted in‐person in a separate mobile unit near the parked mobile medical unit with an outreach specialist present alongside the interviewer. The interviews were led by a sociologist with expertise in qualitative methods or a psychiatric pharmacist working under his supervision. Interviewers followed the question guide but were permitted to pose relevant follow‐up questions to further elucidate the participant's response. Interviews were concluded due in part to data saturation [29] and time constraints related to the funding period. All interviews were recorded by the interviewer with participant consent on a portable recording device, transcribed verbatim by a transcriptionist, and deidentified by the primary researcher to remove personal names and geographic locations. Demographic information was collected by verbally asking each participant at the conclusion of the interview.

We used an inductive approach [30] to analyzing transcripts using Dedoose qualitative analysis software (10.0.33/10.0.34) [31]. Rather than employing a specific determinant framework, the inductive analytic approach was utilised to allow the researchers to remain responsive to participant narratives and avoid constraining findings within predetermined theoretical categories. One researcher reviewed all transcripts and developed a preliminary codebook based on the data. Three researchers then independently coded the same two transcripts to ensure consistent code application and adjusted the codebook to address inconsistencies and gaps in coding through discussion. After finalisation of the codebook, each transcript was independently coded by two of the three researchers and an additional meeting was held to evaluate intra‐coder and inter‐coder reliability over time. The three aforementioned researchers that participated in coding grouped codes into themes by first individually analyzing the data through word‐based techniques, presenting their initial individual findings to the other coders, and then noting overlapping and unique themes between coders. Similar themes were solidified into one overarching theme and unique themes were examined one by one for validity. Group consensus was used to finalise the list of themes. The researchers then developed definitions for each theme and delineated sub‐themes as appropriate. The primary phenomenon of interest was a description of perceived benefits, barriers, and facilitators to accessing mobile OUD services by people involved with the CLS. This study was approved by the Institutional Review Boards of the UIC and Chestnut Health Systems.

## 3 Results

A total of 13 interviews were conducted, ranging in duration from 10 to 33 min (mean of 17 min and 58 s). Over half of the participants identified their gender as male (61.5%), with a mean age of 48 years (SD 11). A majority of participants (61.5%) self‐identified as Black or African American, while 23.1% identified as Caucasian. The remaining two participants selected ‘other’ and no participants identified with a Hispanic or Latino ethnicity. Over half (53.8%) of participants reported they were housed in some capacity in the 30 days prior to the interview, with 38.5% reporting they were unhoused, staying outdoors, in train stations, or shelters. One participant reported spending a majority of the past 30 days detained in jail. Housing status was collected to understand the stability of those visiting the mobile unit. The demographic data was collected in alignment with standards of federal reporting to the National Institute of Health (NIH), the funding source of this study. (Table 2). All participants were either arrested, booked, or charged; released from prison, jail, or electronic monitoring; or currently on probation, parole, or supervised release in the 90 days prior to the interview.

**Table 2:** Interview demographic guide.

What is your age?
What is your gender identity?
Male
Female
Non‐binary/third gender
Some other gender (please provide)
What races, ethnicities, nationalities, or tribes best describe you? (check all that apply)
African American/Black
Caucasian/White
Asian
Hispanic, Latino, Chicano, or LatinX
Alaskan Native
Native Hawaiian
Pacific Islander
Some other group (please provide)
In the past 30 days, where have you been living most of the time?
Shelter (safe havens, Transitional Living Centre [TLC], low‐demand facilities, reception centres, other temporary day or evening facility)
Street/outdoors (sidewalk, doorway, park, public or abandoned building)
Institution (hospital, nursing home, jail/prison)
Housed
◦ Own/rental apartment, room, trailer, or house
◦ Someone else's apartment, room, trailer, or house (including couch surfing)
◦ Dormitory/college residence
◦ Halfway house or transitional housing
◦ Residential treatment recovery residence/sober living
◦ Housed

Five themes and eight sub‐themes emerged related to the experiences of those with recent CLS involvement seeking MOUD at the COIP mobile medical unit. The identified themes, sub‐themes, and representative quotes are compiled in Table 3.

**Table 3:** Emerging themes and related quotes (*n* = 13).

Theme	Sub‐theme	Related quote
1. The mobile unit is a vital service to an underserved and under‐resourced geographic area	Not applicable	*“The outreach you guys are doing is a necessity, especially in impoverished neighborhoods like this” (26, Other, male, outdoors)*.
2. The care environment positively contributes to care utilisation	2.1) The care team culture attracts and furthers engagement	*“They were courteous, they were eager to help, they were very respectful, and whatever you needed, they were there for you.” (59, African American or Black, male, outdoors)*.
	2.2) The mobile unit setting is preferred over other healthcare settings	*“Quicker and they don't judge… they have more compassion over here at the mobile unit than they do in a lot of places.” (40, Caucasian, female, incarcerated)*.
3. The CLS has varied impacts on MOUD care	3.1) Positive assistance from law enforcement	*“Some officers care, put it that way. Some care, care that you're using and they try to get you off. They try to give you resources,” (50, African American or Black, male, outdoors)*.
	3.2) Neutral impact of the CLS	*“Law enforcement? They don't play a part.” (60, African American or Black, male, housed)*.
4. Logistic aspects of receiving MOUD both facilitate and inhibit receiving care	4.1) Mobile unit service characteristics facilitate care	*“I find it very helpful that they give you Suboxone, because they don't, you don't have to wait for a prescription, they give you a supply until you can get your prescription.” (42, Caucasian, female, housed)*.
	4.2) Physically getting to care at the correct time is a barrier	*“Trying to come up with money, excuse me, to catch the bus, you know, just to get over here… it can be hard for a lot of people.” (59, African American or Black, female, housed)*.
5. There are numerous motivators for seeking care at the mobile unit	5.1) Community word‐of‐mouth	*“That's what brought me down here now. We share information with each other. Homeless look out for each other.” (56, African American or Black, female, outdoors)*.
	5.2) Co‐utilisation of services	*“Me and my partner, she and I came here together, so we're supporting each other as far as coming here and getting this done.” (50, African American or Black, male, outdoors)*.
	5.3) Intrinsic self‐motivation	*“I would say my desire to get clean and, you know, take the steps that's necessary for me to achieve that goal [motivates me].” (26, Other, male, outdoors)*.

### 3.1 Theme 1. The Mobile Unit Is a Vital Service to an Underserved and Under‐Resourced Geographic Area

Participants characterised the mobile services as essential to meeting the needs of the region, which has limited access to traditional care. One noted, “*The outreach you guys are doing is a necessity, especially in impoverished neighborhoods like this with a lot going on as far as it being an open‐air drug market.” (26, Other, male, outdoors)* Another participant highlighted the willingness of the team to provide the service to the community. *“You don't find very many people that help the people on the West Side. As you can see, they're tearing down all the grocery stores and stuff. Save‐a‐Lot's the only grocery store left. They said they were shutting down just to remodel it. Couple days later, it was gone.” (40, Caucasian, female, jail/prison)*

### 3.2 Theme 2. The Care Environment Positively Contributes to Care Utilisation

Participants described their encounters with the physical and cultural aspects of the mobile medical unit with high regard. They mentioned that welcoming staff, ample privacy, and individualised attention to their care made them feel respected and valued.

#### 3.2.1 Sub‐Theme 2.1. The Care Team Culture Attracts and Furthers Engagement

The participants overwhelmingly praised the efforts, attitudes, and quality of care provided by the mobile unit staff, noting positive traits such as respect, compassion, and humility contributed to them returning for services. “*I thought the staff was humble… humble is something that your action is. It's not necessarily an adjective or whatever. It's an action, you know, you'll see it, you'll feel it. And I see it and I feel it with you guys.” (44, African American or Black, male, housed)* The care environment was consistently recognised as free from stigma or judgement; traits which were attributed to creating a clinical setting where patients felt comfortable openly discussing their medical histories, needs, and concerns. Said one participant,

It's vital. It's vital for me because I haven't found any program that has made me feel comfortable, comfortable enough to let them know, like, some of my secrets and some of the stuff that has happened to me since I've been using. So, I don't trust anybody else.

(42, Caucasian, female, housed)

The vast array of services available at the mobile unit (including MOUD, harm reduction supplies, wound care, referrals to primary care or mental health providers, and clothing, food, or housing assistance) demonstrated to participants significant kindness and the genuine interest the mobile unit staff have for the community. Notably, participants felt that the effort put forth by the providers demonstrated a willingness and dedication to help that they do not often feel in other service settings in part due to stigma related to their substance use disorder diagnoses.

They were courteous, they were eager to help, they were very respectful, and whatever you needed, they were there for you. To referrals or whatever. As a matter of fact, that's why I'm back this summer.

(59, African American or Black, male, outdoors)

They're very supportive. They don't judge you and they actually listen to what you have to say. They don't just blow you off and be, like, ‘oh, you're a drug addict, it's okay, we're just going to give you this and this and send you on.’ No, they actually ask, ‘Do you need anything else? Is there anything else we can help you with?’ Lots of people don't do that when you're a drug addict.

(40, Caucasian, female, jail/prison)

Specific positive experiences enhanced participant's trust in the mobile services and prompted them to return for continued care. These experiences include successful treatment of a spider bite and subsequent wound, consistent access to buprenorphine leading to reduced opioid use, and encouraging words when going through a challenging period. One participant attributed the open‐door, low‐threshold care model to their continued utilisation of services, stating:

I was really, really sick one day. I had just gotten out of jail, too. So, I was even more sick. Then I came here, and I didn't think I was going to get services. I didn't think I was going to get services at all. And they were, like, ‘Come on.’ I thought because I'd been here before, but I didn't follow up, that they would turn me away. And they're like, ‘No, we get that all the time.’ So, they made me feel a lot more comfortable.

(42, Caucasian, female, housed)

One participant expressed appreciation for the way the mobile unit providers collaborate with patients, saying, “*You guys know in depth what's going on…we go on your recommendations on certain medication when you give us the option to pick what's good for us.” (60, African American or Black, male, housed)* In addition to highlighting the importance of provider/patient collaboration, participants felt that the team genuinely “*sees them at their level” (56, African American or Black, female, outdoors)* and possesses a keen knowledge of how to provide care to people with unstable housing. Participants felt respected when the staff take time to explain medical situations and answer all questions. They endorsed welcoming care from every team member, noting that it was especially encouraging to interact with staff with lived substance use experience.

We all came over here and we started talking to the people and we found out that, hey, they changed their life. They were addicts just like we were. Or at least they had the background of being professionals and being down to earth… They were on our level. The people are wonderful a lot of them are recovering addicts, they come out the van and really talk to you.

(59, African American or Black, male, outdoors)

#### 3.2.2 Sub‐Theme 2.2. The Mobile Unit Setting Is Preferred Over Other Healthcare Settings

Participants consistently expressed a preference for the care provided on the mobile medical unit over other healthcare settings, especially the emergency room. The efficiency, convenient location, and personability of the mobile unit staff were all factors influencing these emerging preferences.

The people are very, very, like I said, very personable. Although they have to be getting in your business a little bit, they make you feel comfortable being open to them. So, that's a good deal. I'm more open here probably than with a doctor in an emergency room visit.

(50, African American or Black, male, outdoors)

Instead of going to a hospital, okay, you have to sit up there for hours and the more you sick, the sicker you get, the worse you get. With you guys, they just want to know your address, do you have any medical conditions, are you allergic to certain medications, and then they give you like, a week supply.

(60, African American or Black, male, housed)

The convenience is there's a lot of us out here. Number one, it's a lot of us out here scared to go to the doctor, number one. Number two, they're afraid of what they're going to find out about their body, about themselves. You should want to know about yourself. Being that they're here every certain days, on the mobile van, that's your chance right there to want to save your life.

(59, African American or Black, female, housed)

The aforementioned lack of judgement was a notable benefit of the mobile unit compared to other healthcare experiences. The mobile unit was described as more welcoming and comfortable, with fewer barriers to care. Participants thought the mobile unit model of on‐site buprenorphine dispensing and walk‐in appointments was better suited to meet the needs of people with substance use disorders and retain them in care.

They differ because it's not a lot of red tape or paperwork. They're immediate action. Whatever you need, they take care of it right there. If you need to see somebody in the van, they're there. The wait times aren't that long because they're here for so long. So, if you go to a doctor's office, or a rehab center, or go to try to get Suboxone, or any Methadone, it's a process that just, for us, being addicts, man, it just takes too long. Cause half the people walk out.

(59, African American or Black, male, outdoors)

Quicker and they don't judge. They don't judge you. I've been to many doctors’ offices and I stopped going because they judge you because you're a drug addict. And I got tired of people looking at me crazy and talking crap to me. Well, you need to do this and you need to do this. Well, you don't know what I'm going through. So, they have more compassion over here at the mobile unit than they do in a lot of places.

(40, Caucasian, Female, jail/prison)

### 3.3 Theme 3. The CLS Has Varied Impacts on MOUD Care

There was a range of perceptions and experiences pertaining to the CLS and its impact on access to MOUD care. Participants perceived stark differences among police officers’ goals; noting that some officers sought to help those with OUD connect to treatment, while other participants perceived officers as prioritising punitive action in the form of arrests. Of the single participant to report jail as their place of residence in the 30 days prior to the interview, they noted a lack of access to MOUD services, both generally and from the mobile unit, as they did not receive MOUD while in jail. They described their desire to consistently pursue care with the mobile unit if they were able, and immediately sought mobile MOUD services as soon as they were released from jail.

They hindered me from being able to use it ‘cause I got arrested the last time. I came here, I was supposed to come back, I came here, I think it was a Monday, and I was supposed to come back that Friday, but I ended up getting arrested the next day. And I was in there for a while…I wasn't able to come back to get the actual prescription… [the jail] pretty much told me I had to go cold turkey. Yeah, I was sick. It was kind of bad.

(40, Caucasian, female, jail/prison)

#### 3.3.1 Sub‐Theme 3.1. Positive Assistance From Law Enforcement

Some participants felt that law enforcement officials contributed positively to accessing MOUD at the mobile unit. They described the officer's desire to help people stop using substances and the lack of interference when participants approached the mobile unit for services. There was no mention of services or resources offered to those held within jails or prisons.

Some officers care, put it that way. Some care, care that you're using, and they try to get you off. They try to give you resources.

(50, African American or Black, male, outdoors)

The law enforcement encourage us to come to the mobile unit.

(56, African American or Black, female, outdoors)

#### 3.3.2 Sub‐Theme 3.2. Neutral Impact of CLS

For many participants, local police officers neither interfered with nor promoted access to mobile MOUD services, thus having a neutral effect on care utilisation. The same was described for parole officers.

Being a cop, you're going to ride past. You ain't done nothing wrong, they ain't going to bother you. But the police not going to stop and say, hey, you want to use the mobile unit? They don't do that.

(64, African American or Black, male, housed)

There was a notable mentality of autonomy to seek care with no thought given to the opinions or actions of the CLS.

It doesn't affect me at all.

(52, African American or Black, male, housed)

I wouldn't say it affects [my ability to use the mobile unit].

(29, Other, male, outdoors)

None, they don't affect me. I do what I want to do.

(44, African American or Black, other, male, housed)

Law enforcement? They don't play a part.

(60, African American or Black, male, housed1)

When asked about the impact of arrest and jail detainment, one participant described periods of incarceration short enough not to impact care, in part because of the consistent mobile unit schedule.

At the most, I'll spend a couple days, like last week, spent a couple days, and then they let me out. And the mobile unit's always here, so.

(59, African American or Black, male, outdoors)

### 3.4 Theme 4. Logistic Aspects of Receiving MOUD Both Facilitate and Inhibit Receiving Care

Participants reported that numerous mobile unit locations (which rotate throughout the week) improved access to care since they could present to different locations on different days as needed. They found the locations to be conveniently situated throughout the most active drug markets in the city and near where many of them resided. The consistent outreach hours every day of the week made it simple to know when to seek care, with one participant saying, “*You guys are always very structured. I mean, you make it hard to not be on time.” (29, Other, male, housed)*

The no‐cost model of providing care was a catalyst for participants to become involved with services and ultimately receive referrals to other clinics.

The initial thought and concern about this mobile vehicle being here is they help individuals that need help and can't afford to get out there and get the help that they need. They need someone to help them with the follow‐up and to go get to help. So that they be able to get help from starting here until they learn to where they can start themselves.

(59, African American or Black, female, housed)

A consistent suggestion for improvement was to expand hours, both earlier and later in the day, and adding weekend outreach days to enhance access to care.

There were days where they wasn't here and you wished they would, they were here.

(59, African American or Black, male, outdoors)

I know everybody needs a day off, but we actually look forward to the trucks being here. And when they're not here, I'm sad.

(56, African American or Black, female, outdoors)

Despite the mobile van's consistent schedule, some participants experienced challenges. Numerous participants shared instances of arriving for a walk‐in appointment too late in the day and being asked to return at a later date because the clinic was full. While some participants reported the flyers given to them at the end of their visits were helpful in keeping them timely, others stated that lack of awareness of the schedule made it difficult for them to present for care.

I didn't have the paper before so I didn't know what times, and stuff like that. So, I usually, sometimes I would miss them. When I got out, I tried to come down here but I couldn't remember what days you guys were on [street name]…

(40, Caucasian, female, jail/prison)

#### 3.4.1 Sub‐Theme 4.1. Mobile Unit Service Characteristics Facilitate Care

Participants identified multiple characteristics of the mobile unit services that made receiving care straightforward, including walk‐in appointments and on‐site dispensing of buprenorphine. They appreciated the minimal wait time, noting that the mobile unit's practices align well with the needs of people who use drugs.

I find it very helpful that they give you Suboxone, because you don't have to wait for a prescription. They give you a supply until you can get your prescription. And that's what I like about it.

(42, Caucasian, female, housed)

That's what addicts look for. We tired of all that rigmarole and all that red tape. We're looking for a fast service, convenient. Because we want what we want when we want it.

(59, African American or Black, male, outdoors)

The wraparound services such as wound care, primary care, food and clothing donations, and referrals to services beyond the abilities of the mobile unit positively influenced participants to return for care. They appreciated being able to be seen for a spectrum of medical needs at one clinic and noted that the array of services as a positive factor in favour of the mobile unit over other healthcare venues, as discussed in sub‐theme 2.1.

When I first came here, I was surprised, and I liked it, how they can put so much in one little truck. I like it. Yeah. It's very, very, accessible.

(52, African American or Black, male, housed)

The participants are comfortable returning for care at the mobile unit in part due to the consistent workflow and quality of care they receive. Specifically mentioned was the comprehensive counselling they received during a clinical encounter, which answered all their questions, provided clear instructions, and made knowing how and when to follow‐up simple.

#### 3.4.2 Sub‐Theme 4.2. Physically Getting to Care at the Correct Time Is a Barrier

In contrast to some of the praise for the specific locations of the mobile unit, numerous participants described challenges reaching the sites because of reliance on public transportation. They described both timing challenges and difficulty paying for transit fare. One patient noted that they had to specifically prioritise spending on bus fare in order to make it to the mobile unit for MOUD.

Trying to come up with money to catch the bus just to get over here… it can be hard for a lot of people.

(59, African American or Black, female, housed)

I catch the L or I take the CTA bus and, you know, I don't always have the fare for each and every bus and, you know, train or something like that.

(26, Other, male, outdoors)

### 3.5 Theme 5. There Are Numerous Motivators for Seeking Care at the Mobile Unit

Numerous factors emerged as motivators or facilitators to care‐seeking behaviour including intrinsic motivation and perceived readiness‐for‐change and external influences such as friends or family members.

#### 3.5.1 Sub‐Theme 5.1. Community Word‐of‐Mouth

Community word‐of‐mouth was noted as an essential facilitator for seeking care at the mobile unit. Many participants reported that their friends and family members encouraged them to engage with mobile services.

That's what brought me down here now. We share information with each other. Homeless look out for each other.

(56, African American or Black, female, outdoors)

My family, they pushes me. I come from a very close‐knit family and they tell me, go on that mobile van and get a head start with whatever it could be. Get a head start and be able to get rid of it. And that's a good thing to come out there.

(59, African American or Black, female, housed)

Additionally, participants said that resources within the community supported and motivated them to seek care at the mobile unit.

The community, they tell us what the truck's for and we check it out. So, the community do and churches do help out and let us know what type of truck it is.

(52, African American or Black, male, housed)

The library staff lets us know. You can go, there's a truck there on Fridays and there's days you have to be here and they'll help with that, the people will help you.

(56, African American or Black, female, outdoors)

One mentioned that their programme supported them financially by facilitating transportation to the outreach location. *“I go through a program…and they help me with rides and stuff if I need to get rides or they'll get me bus cards… So, that's a good thing. I really don't have to worry about transportation no more because of that program.” (40, Caucasian, female, jail/prison)* Another noted that their diversion programme, a form of pretrial sentencing, referred them to the programme. *“I got arrested. So, because I didn't have no warrants, they were able to let me do the diversion program and the counselor is the one who told me about the mobile unit.” (42, Caucasian, female, housed)*

#### 3.5.2 Sub‐Theme 5.2. Co‐Utilisation of Services

The connectedness among people who use drugs plays a critical role in being informed about and encouraged to engage with services. Participants expressed that accessing the mobile services together was motivational, often citing coming with partners or friends as support.

Me and my partner, she and I came here together, so we're supporting each other as far as coming here and getting this done.

(50, African American or Black, male, outdoors)

One of my friends brought me here today. Being around addicts, we tell each other about good things that are happening. And everybody around here that uses drugs knows about it. This has been here so long, everybody knows about it. So, you know, we support each other in that fact.

(59, African American or Black, male, outdoors)

#### 3.5.3 Sub‐Theme 5.3. Intrinsic Self‐Motivation

Some participants identified their drive to achieve their recovery goals as an intrinsic motivator to access mobile services. *“I would say my desire to get clean and, you know, take the steps that's necessary for me to achieve that goal [motivates me].” (26, Other, male, outdoors)* Reaching a critical low point in their substance use trajectory increased people's motivation to engage with mobile services.

You get tired of putting [drugs] in your system… and sometimes you get to the point in life, I don't want to do this anymore… That's where coming here makes a difference. Because you can talk to somebody about how you feel… that's the bottom line to it. If you want to quit using… and you want help, it's up to you to do it. Ain't nobody going to do it for you. So, it's up to you to walk through that door. But I can say that if you walk through that door, they're there with you 100%.

(59, African American or Black, male, outdoors)

## 4 Discussion

In this study of people recently involved with the CLS accessing addiction treatment services at a mobile medical unit, participants emphasised the value of low‐threshold, non‐judgmental MOUD services and contrasted their experiences with traditional healthcare settings. They downplayed the impact of law enforcement on their ability to seek services in the community but described how incarceration impedes access to MOUD while incarcerated. This is consistent with data that shows that less than half of US jails offer MOUD, and only 12.8% offer MOUD to all people with OUD. Significant regional variation exists across the United States, affecting access, with fewer jails in the South and Midwest reporting MOUD provision compared to those in the Northeast [32]. This study amplifies the voices of people with varying levels of recent CLS involvement, housing status, age, and experiences receiving mobile medical care. It accurately reflects the average demographics of patients cared for by the same Chicago mobile unit and similar units nationwide [19, 20].

A consistent sentiment amongst study participants was the preference for the mobile unit over other healthcare settings. The stigma‐free language and behaviours of clinical staff greatly impacted participant satisfaction and informed their choice to return for follow‐up care at the mobile unit and encourage other members of their community to access services. These findings align with prior work demonstrating that poor treatment, including dehumanising language, discriminatory decision‐making, and undertreatment of withdrawal can delay or prevent presentation for care and potentially lead to early hospitalisation self‐termination [33, 34]. Stigma is a particularly salient issue when working with people with recent CLS involvement, given the well‐documented prevalence of stigmatising views towards OUD throughout the CLS which has hindered implementation of treatment more broadly [12]. People with CLS involvement may feel heightened concerns about sharing details related to substance use in any setting, including when seeking healthcare. The presence of police in healthcare settings such as the emergency department could lead to patients feeling diminished trust of their provider and experiences of privacy breaches, which may be of particular concern to those with CLS involvement [35]. Low‐barrier mobile programmes that facilitate open communication may mitigate this concern, allowing for more responsive care. For example, DEA regulations require the presence of security personnel on vehicles storing and dispensing controlled substances, such as buprenorphine. To comply with these regulations and simultaneously reduce the intimidating nature of uniformed officers, the COIP Mobile MOUD programme encourages security personnel to dress in casual clothing as opposed to overt security uniforms, thus enhancing the approachable, community‐oriented atmosphere.

Many participants also noted that the workflow of the mobile unit, particularly low wait times and on‐site dispensing of MOUD, was preferred over standard workflows of emergency rooms or opioid treatment programmes. Though the participants were not explicitly asked about their insurance status, it has been shown that people with demographics similar to this study lack active insurance or are unable to confirm insurance status at the time of visit [19]. Inconsistent or non‐existent insurance may strongly dictate the settings available for people to receive care, such as frequently utilising emergency rooms versus regular appointments with a primary care provider. Re‐entry from jail or prison is a well‐recognised time during which people experience insurance gaps that limit their ability to access care. Despite recent efforts by the Centres for Medicare and Medicaid Services to address this care gap, insurance status remains a critical issue during the re‐entry time period [11]. Burns et al. recently examined the impact of a pre‐release Medicaid enrolment programme on healthcare utilisation among adults with a history of substance use disorder; notably, although the programme was associated with an increase in post‐release outpatient care, there was no change in hospital‐based service utilisation, suggesting that additional support is needed to connect people with substance use disorder to care post‐release [36]. Given the significant increase in overdose mortality after release from jail or prison, this is a critical window to ensure access to immediate OUD treatment, regardless of insurance status [16, 17, 18]. The trust built toward a consistent, stigma‐free mobile unit placed directly in the community that can provide immediate, same‐day treatment initiation can facilitate care for those unable or unwilling to present elsewhere, filling gaps in insurance coverage and dignified healthcare.

This study highlights the mixed impact that law enforcement, parole officers, and correctional staff may have on engagement with SUD treatment. It is not surprising that the CLS is not necessarily perceived by those with OUD as a potential support to their recovery. A recent survey of jails in counties highly impacted by the opioid epidemic found that while many had MOUD available in‐house, only 20% provided it to *anyone* with an OUD diagnosis or provided a bridge supply at release [37]. This is a significant missed opportunity for engagement and initiation of treatment. Recent studies suggest that educational initiatives can improve correctional officers’ opinions of MOUD, which may lead to increased connection to care [38]. These types of initiatives are needed across multiple settings in which people engage with law enforcement and the CLS. A 2023 survey of Illinois police officers found that officers held stigmatising views towards people with OUD [39]. Initiatives to improve attitudes towards MOUD would likely facilitate improved referrals and connections to treatment from law enforcement. Collaboration across the CLS, local law enforcement, and low‐barrier mobile units could streamline treatment initiation, prevent post‐release gaps in care, and foster a more supportive rather than punitive environment for people with OUD in the community.

Participants were given the opportunity to recommend areas of improvement for the programme. They emphasised the value of peer support and community engagement as key recovery components, especially for those involved in the CLS. Participants recommended that the mobile unit increase the presence of peer support specialists, host regular community meetings or support groups in public spaces, such as libraries, and involve people with lived experience of substance use in the care team. COIP's use of the Indigenous Leader Outreach Model (i.e., employing community members with lived experience to provide services and conduct research) already reflects a culturally grounded approach to medical outreach that centres community trust with peer leadership and aligns with participant‐driven suggestions [26]. There is a growing body of research emphasising that peer support in substance use disorder care enhances treatment engagement and medication initiation, reduces overdose, and builds sustained connections throughout the recovery continuum [40, 41]. Multiple engagements with peer support specialists delivering harm reduction have been linked to improved housing and criminal justice status [42]. These findings underscore the perceived and known benefit of human connection on recovery and will be further explored by the mobile unit to enhance their relevance and impact on those recently involved with the CLS.

In recognition that workflow characteristics such as low wait‐time, walk‐in appointment, and on‐the‐spot buprenorphine dispensing are crucial to participant retention, the mobile unit staff will monitor the need for additional clinicians and expanded units to maintain efficiency, with the potential to expand services to a vehicle specifically targeting those with recent CLS involvement. Positioning a low‐barrier unit directly outside of an urban jail has been shown to facilitate immediate access to buprenorphine, bridging the treatment continuity gap, especially for jails that do not provide medication during a person's time in custody or at release [20]. Establishing a presence near a jail, enhanced re‐entry support, and establishing inner‐jail collaboration could prove to be lifesaving in this phase of high overdose risk. Though a known barrier, it is paramount for the CLS to invest in supporting people who use drugs through inner‐agency communication and coordination of pre‐release planning and treatment gap provisions [43].

While the CFIR framework was utilised to develop the interview question guide, no theoretical framework was employed to lead analysis [44]. This decision was made to allow the researchers to remain open to identifying unique themes and sub‐themes during their inductive analysis [30]; a pragmatic approach selected to fit the needs of the project. The results of this qualitative study are in alignment with pre‐existing theoretical frameworks, including CFIR and the patient‐aspect of the Practical, Robust Implementation Sustainability Model (PRISM) [44, 45]. Participants’ comments aligned with multiple CFIR constructs, including innovation relative advantage and source, local conditions and attitudes, human equality and recipient centeredness, compatibility, space, and tension for change. Additionally, the interviews resulted in findings related to the competing demands, organisational health and culture, service and access, burden, patient centeredness, and patient choice, all of which are elements within PRISM. These findings highlight and improve on the theoretical generalisability of such frameworks, which are foundational pillars of implementation science. This alignment adds to the body of work positively supporting methods for bringing definitions and clarity to dynamic and complex human processes [46]. The study findings additionally agree with a portion of results from a 2025 scoping literature review aimed at exploring the preferences and attitudes toward MOUD of people with legal involvement, having both found negative opinions of traditional treatment settings and an intertwining between structural barriers influencing personal preferences and attitudes [47].

The findings of this study are limited to the experiences and opinions of people accessing MOUD services in a few neighbourhoods on the West Side of Chicago. This study was limited in recruitment capacity due to its funding timeframe, interviewer availability, and recruitment period co‐occurring with the slower (winter) months of COIP mobile unit outreach. As such, the number of people experiencing the various types of CLS involvement was limited. Future research developed to explore the experiences of people with CLS involvement who cannot access traditional clinical or mobile services would inform efforts to make addiction treatment services equitable and accessible to all. It would additionally be beneficial to conduct interviews to assess people's experiences with OUD care within jails and prisons. An enhanced understanding would be gained through expanded recruitment throughout additional neighbourhoods where people involved with the CLS live and seek care. Despite the limited geographic reach, the use of community voices in any capacity enhances the validity of the data. Further research should evaluate differences between the opinions and experiences of those detained in jail versus incarcerated in prison, which was not overtly collected in this study.

## 5 Conclusions

This single‐centre, qualitative study showed that people with recent CLS involvement are seeking mobile services to access low‐threshold buprenorphine, harm reduction supplies, and comprehensive medical care. They favour walk‐in, stigma‐free, and compassionate care in the mobile setting over other traditional healthcare settings. Though the participants expressed a range of opinions regarding the impact of the CLS on accessing mobile services, it is important to acknowledge the significantly limited access to MOUD directly in jails and continue to advocate for increased availability of MOUD, especially at the time of release. Participants identified a number of barriers to MOUD access (stigma, lack of peer support, and unstable housing) that, while not directly tied to the CLS, are disproportionately prominent challenges for people with CLS‐involvement. Disparities in MOUD access highlight the importance of reaching people with OUD reintegrating into communities immediately upon release as a modality of overdose prevention. Additional services are required to tailor to this population, including broader implementation of re‐entry support such as peer recovery and recovery‐oriented support groups.

## Author Contributions

**Toni Martinford:** writing – original draft preparation, investigation, formal analysis, visualisation, project administration. **Dennis P. Watson:** writing – review and editing, conceptualisation, methodology, funding acquisition. **Sarah Messmer:** writing – original draft preparation, writing – review and editing, resources. **Leyla Rashid:** formal analysis. **Monte Staton:** investigation. **Michael L. Dennis:** conceptualisation, methodology, funding acquisition. **Christine E. Grella:** writing – review and editing, conceptualisation, methodology, funding acquisition. **Abigail Elmes‐Patel:** writing – original draft preparation, writing – review and editing, formal analysis, supervision.

## Disclosure

All opinions expressed are the authors’ and do not necessarily represent those of the funding agency.

## Conflicts of Interest

The authors declare no conflicts of interest.

## Data Availability

The data that support the findings of this study are available from the corresponding author upon request.
